# Pharmacodynamic interaction of fenugreek, insulin and glimepiride on sero-biochemical parameters in diabetic Sprague-Dawley rats

**DOI:** 10.14202/vetworld.2015.656-663

**Published:** 2015-05-26

**Authors:** C. Haritha, A. Gopala Reddy, Y. Ramana Reddy, B. Anilkumar

**Affiliations:** 1 Department of Pharmacology, College of Veterinary Science, Sri Venkateswara Veterinary University, Korutla, Telangana, India; 2 Department of Pharmacology, College of Veterinary Science, Sri Venkateswara Veterinary University, Rajendranagar, Hyderabad, Telangana, India; 3 Department of Animal Nurtition, College of Veterinary Science, Sri Venkateswara Veterinary University, Rajendranagar, Hyderabad, Telangana, India

**Keywords:** cholesterol, diabetes, fenugreek, glimepiride, glucose, insulin, protein

## Abstract

**Aim:**

This study was undertaken to assess the pharmacodynamic interaction of fenugreek, insulin and glimepiride on sero-biochemical parameters in streptozotocin-induced diabetic rats.

**Materials and Methods:**

A total of 56 male Sprague-Dawley rats, randomly divided into seven Groups. Group 1: Non-diabetic control; Group 2: Streptozotocin induced diabetic control; Groups 3, 4 and 5 were treated with insulin, glimepiride and fenugreek seed powder, respectively; Groups 6 and 7: Insulin + fenugreek seed powder treatment and glimepiride + fenugreek seed powder treatment respectively, in diabetic rats. Body weights, blood glucose, lipids total cholesterol (TC), high-density lipoprotein (HDL), low-density lipoprotein (LDL), triglycerides (TG) and proteins (total protein, albumin, globulin, A/G ratios) were studied at different time intervals. Rats were sacrified at the end of 8 weeks, pancreas and aorta collected for histopathological study.

**Results:**

The results of Group 2 showed significantly (p<0.05) higher concentration of glucose, TC, TG, LDL, globulin, A/G ratios and significantly (p<0.05) lower concentration of albumin, total protein, HDL and body weights when compared to Group 1 at the end of 4^th^ and 8^th^ weeks intervals with marked alteration in histopathology of pancreas and aorta. All the treatment Groups 3-7 showed significantly (p<0.05) improvement in the all the parameters and the Groups 6 and 7 showed highest decrease in the concentration blood glucose, TC, TG, LDL and increase in the albumin, total protein and body weights during 6^th^ and 8^th^ week, respectively.

**Conclusion:**

The treatment with fenugreek, insulin and glimepiride countered the alteration in the sero biochemical parameters in diabetic rats, and their combination was found a positive interaction in improving the sero biochemical status of diabetic rats.

## Introduction

Diabetes mellitus (DM) is a syndrome, characterized by hyperglycemia, altered metabolism of lipids, carbohydrates and proteins, and an increased risk of complications from vascular disease [1]. The present strategy for treating DM Type-1 and most Type-2 is insulin therapy and oral hypoglycemics are used to treat Type-2 DM, which includes, biguanides (e.g.; metformin), thiazolidinediones (e.g.: Rosiglitazone, pioglitazone); a-glucosidase inhibitors (e.g.; acarbose) and insulin *secretagogues:* Sulfonylureas (tolbutamide, chlorpropamide, *gliclazide*, glimepiride). These drugs could not reinstate a normal euglycemic pattern whether used alone or in combination and whether administered as a regular or intensive regime [2,3]. However due to unwanted side-effects and low efficacies of these drugs, plants have been suggested as a rich and yet unexplored alternatives of potentially useful anti-diabetic drugs. Moreover, in general, practice, the oral hypoglycemics are used along with the herbs in several cases [4].

Fenugreek (*Trigonella foenum-graecum*) is known to have several pharmacological effects such as hypoglycemic [5], hypocholesterolemic [6], antioxidant [7], appetite stimulation, regresses pre-established cholesterol gallstones [8]. Glimepiride, a second generation sulfonylurea agent for Type-2 DM, lowers blood glucose by stimulating the release of insulin from functioning pancreatic beta cells and has antioxidant and hypolipidemic actions [9]. Most of the herbal remedies can interact with allopathic drugs resulting in altered activity and toxicity. Current published/research information on herb-drug interactions is scanty.

Hence, the present experimental study was planned to evaluate the pharmacodynamic interaction of fenugreek seed powder with insulin and glimepiride in diabetic Sprague-Dawley rats.

## Materials and Methods

### Ethical approval

The experimental protocol was approved by Institutional Animal Ethics Committee (approval no. 6/I/10).

The experimental study was conducted on male Sprague-Dawley rats of uniform age (3 months) and weight that were procured from National Center for Laboratory Animal Sciences, National Institute of Nutrition (NIN), Hyderabad.

### Materials

#### Drugs

1. Streptozotocin (SRL Pvt. Ltd., Mumbai) was dissolved in 0.5 M sodium citrate, pH 4.5
2. Glimepiride (Ranbaxy, India). Glimepiride was administered as suspension in freshly prepared 0.5% w/v carboxymethyl cellulose sodium salt
3. Insulin (Insuman Basal-Aventis).

#### Herb

Fenugreek (*T. foenum-graecum*) seeds were purchased from the local market, shade dried, powdered and administered as suspension freshly prepared in 0.5% w/v carboxy methyl cellulose sodium salt.

#### Chemicals and kits

All chemicals used in the biochemical analysis were of analytical grade.

1. All the chemicals (for preparation of reagents and buffers) were procured from Qualigens Pvt. Ltd., Mumbai and SRL Pvt. Ltd., Mumbai
2. Kits for glucose, total protein, albumin, total cholesterol (TC), triglycerides (TG) and high-density lipoprotein cholesterol (HDL-C) were procured from Span Diagnostics Ltd., Surat, India.

### Methods

#### Animals

A total of 56 male Sprague-Dawley rats of uniform age and weight were procured for the study. Feed (Feed in the form of the pellet as per NIN feed standard) and water was provided ad libitum throughout the experiment. Animals were housed in polypropylene cages in a well-ventilated animal house with 12-12 h light – dark cycles. Acclimatization period of 2 weeks was observed before the start of the experiment.

#### Induction of diabetes and initiation of herb/drug treatment

After an acclimatization period of 2 weeks, rats were randomly divided into 7 Groups of 8 rats in each and blood samples were collected, and serum was separated for glucose estimation. Subsequently, Group 1 was kept as normal control throughout the experimental period. Remaining six groups were induced diabetes by intra-peritoneal injection of streptozotocin @ 40 mg/kg body weight. The rats were provided with glucose water for 24 h to prevent hypoglycemia. Blood samples were collected after 72 h and serum was separated for glucose estimation. Rats with blood glucose value of >250 mg/dl (72 h after streptozotocin administration) were included in this study (n=8). Treatment protocols were initiated from day 2 post-confirmation of diabetes (day 5 post-streptozotocin administration) and were continued for 8 weeks.

#### Experimental design

After induction of diabetes, all the groups were maintained as per the following drug and herb treatment schedule for 8 weeks.

Group 1: Non-diabetic control
Group 2: Streptozotocin (40 mg/kg i/p single dose)-induced diabetic control
Group 3: Insulin (4 U/kg once daily for 8 weeks) treatment in diabetic rats
Group 4: Glimepiride (4 mg/kg orally once daily for 8 weeks) treatment in diabetic rats
Group 5: Fenugreek seed powder treatment (1 g/kg orally once daily for 8 weeks) in diabetic rats
Group 6: Insulin + fenugreek seed powder treatment (once daily for 8 weeks) in diabetic rats
Group 7: Glimepiride + fenugreek seed powder treatment (once daily for 8 weeks) in diabetic rats

#### Blood collection

Blood collection was carried out at every 2 weeks interval for sero-biochemical analysis after initiation of the drug administration till the end of experiment (8 weeks). Feed was withdrawn 12 h before the blood collection and blood was collected through retro-orbital plexus after ether anesthesia into serum vacutainers and centrifuged at 3000 RPM for 15 min and serum was separated and stored at −20°C till analysis. The sera samples were analyzed for the concentration of glucose (every 2 weeks), total protein, albumin, TC, HDL-C and TG on 4^th^ and 8^th^ week and average body weights were recorded at weekly intervals in all the groups. At the end of 8 weeks rats were sacrified, pancreas and aorta collected for histopathological study as per standard protocol [10].

## Results and Discussion

This study result revealed that the body weights in Groups 3-7 were significantly higher as compared to Group 2 at the end of 2^nd^ and 3^rd^ week. Groups 5, 6 and 7 showed significantly highest increase in body weights among all the treated groups at the end of 4^th^, 5^th^ and 6^th^ week. Groups 6 and 7 showed significantly higher body weights among the treated groups at the end of 7^th^ and 8^th^ week (Table-1). The decrease in body weight in diabetic rats is due to insulin depletion resulting in reduced peripheral glucose utilization and excessive breakdown of tissue proteins [11]. There is an increased catabolic response in the liver, adipose tissue and muscle. The decrease in body weight in diabetic rats could be due to dehydration and catabolism of fats and proteins. Increased catabolic reactions leading to muscle wasting might also be the cause for the reduced weight gain by diabetic rats. Glimepiride and fenugreek treatment improved body weights, probably due to increased insulin secretion.

**Table-1:** Weekly body weights of different groups of rats.

Group	0 week	1^st^ week	2^nd^ week	3^rd^ week	4^th^ week	5^th^ week	6^th^ week	7^th^ week	8^th^ week
Non-diabetic control	271.17±5.87^ab^	291.33±5.49^c^	313.67±6.18^d^	334.17±6.30^d^	343.17±6.85^d^	362.00±6.88^d^	376.17±6.19^d^	389.50±5.26^e^	405.33±4.26^e^
DM control	271.50±5.70^ab^	253.50±6.06^a^	234.33±5.75^a^	220.00±6.12^a^	208.67±5.94^a^	200.83±6.69^a^	194.17±6.25^a^	188.33±5.82^a^	181.67±5.64^a^
DM+Insulin	259.00±6.12^ab^	264.00±6.14^ab^	270.50±5.98^b^	277.50±6.21^b^	283.83±6.43^b^	291.83±6.14^b^	299.17±6.37^b^	305.83±6.50^b^	312.00±6.42^b^
DM+GM	270.67±5.17^ab^	275.50±4.95^bc^	283.67±4.69^bc^	292.17±4.85^bc^	301.00±5.13^bc^	308.00±4.92^bc^	315.00±4.61^bc^	320.50±4.65^bc^	328.00±4.52^c^
DM+FG	278.17±5.25^a^	283.33±5.40^bc^	292.67±5.58^c^	300.00±5.44^c^	308.17±5.49^c^	317.00±4.91^c^	322.83±4.65^c^	330.00±4.63^cd^	337.50±4.36^cd^
DM+Insulin+FG	262.83±8.02^ab^	272.67±7.96^abc^	282.67±7.83^bc^	294.67±6.93^bc^	307.67±6.93^c^	319.00±6.65^c^	329.33±5.90^c^	339.00±5.48^d^	351.00±4.97^d^
DM+GM+FG	255.67±8.16^b^	267.50±8.16^ab^	277.50±8.26^bc^	289.33±7.89^bc^	302.50±7.37^bc^	316.00±6.77^c^	326.67±6.57^c^	337.83±6.31^d^	351.00±6.34^d^

Values are mean±standard error (n=8). DM=Diabetic mellitus, FG=Fenugreek, GM=Glimepiride. Means with different alphabets as superscripts differ significantly (p<0.05); Capital alphabets for horizontal comparison and small alphabets for vertical comparison

**Figure-1:**
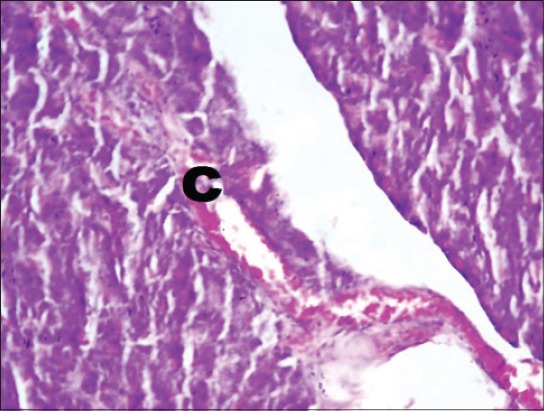
Photomicrograph of pancreas showing marked congestion (C) (H and E, ×200; Group 2).

**Figure-2:**
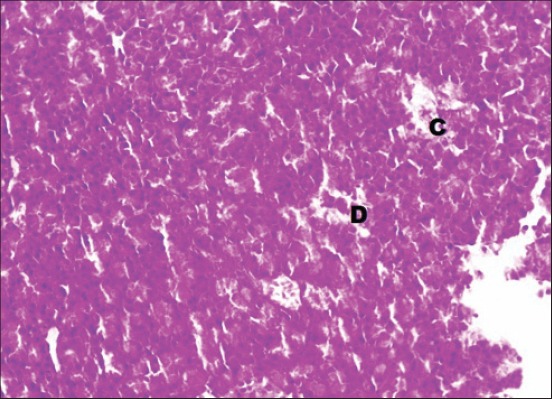
Photomicrograph of pancreas showing mild congestion (C) and depleted acini (D) (H and E, ×200; Group 2).

**Figure-3:**
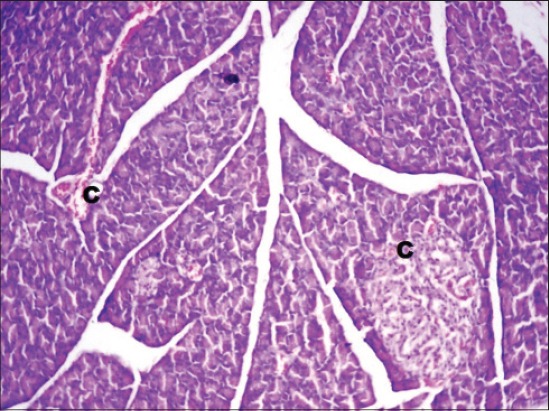
Photomicrograph of pancreas showing moderate congestion (C) (H and E, ×200; Group 4).

**Figure-4:**
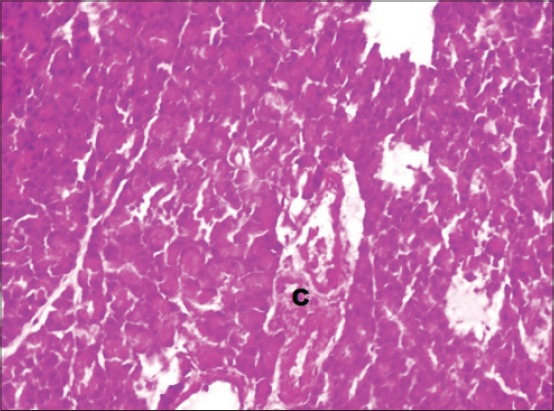
Photomicrograph of pancreas showing mild congestion (C) (H and E, ×200; Group 7).

The serum glucose levels in diabetic control Group 2 were significantly higher as compared to other groups throughout the experiment. All the treated Groups 3-7 showed a significant decrease in glucose concentration during 2-8^th^ weeks as compared to Group 2. The combination Groups 6 and 7 showed highest decreased in blood glucose concentration during 6^th^ and 8^th^ week among all the treated Groups (Table-2). This was further supported by an improvement in the histology of the pancreas. The pancreatic sections of Group 2 showed marked congestion between acini (Figure-1). Few sections showed depleted acini (Figure-2). The Groups 3, 4 and 5 revealed moderate congestion (Figure-3). The Groups 6 and 7 showed mild changes (Figure-4).

**Table-2:** Serum glucose concentration (mg/dl) of different groups of rats.

Group	0 week	2^nd^ week	4^th^ week	6^th^ week	8^th^ week
Non-diabetic control	78.83±6.60^aA^	79.94±2.16^aA^	89.30±3.16^aA^	90.33±2.03^aA^	87.40±1.53^aA^
DM control	342.33±20.67^bA^	390.13±14.93^eB^	427.53±14.77^bB^	568.45±14.41^dC^	421.04±3.95^eB^
DM+Insulin	348.17±17.28^bA^	222.99±3.64^bcB^	219.07±4.10^cB^	215.78±1.98^cB^	210.77±2.75^dB^
DM+GM	330.83±12.78^bA^	228.20±8.74^cB^	219.98±2.67^cB^	216.54±2.17^cB^	206.86±3.17^cdB^
DM+FG	319.83±19.72^bA^	254.19±4.85^dB^	223.71±5.17^cC^	213.36±1.62^cC^	200.40±2.01^cC^
DM+Insulin+FG	323.00±21.27^bA^	202.76±2.24^bB^	207.55±1.77^cB^	190.59±1.30^bB^	181.58±4.02^bB^
DM+GM+FG	335.00±10.38^bA^	213.82±4.60^bcB^	205.64±1.07^cB^	184.22±2.58^bC^	174.16±3.23^bC^

Values are mean±standard error (n=8). Means with different alphabets as superscripts differ significantly (p<0.05); Capital alphabets for horizontal comparison and small alphabets for vertical comparison. DM=Diabetic mellitus, GM=Glimepiride, FG=Fenugreek

Fenugreek seed contains 45-60% carbohydrates, mainly mucilaginous fiber (galactomannans); 20-30% proteins high in lysine and tryptophan; 5-10% fixed oils (lipids); pyridine-type alkaloids mostly trigonelline (0.2-0.36%), choline (0.5%), gentianine and carpaine; flavonoids (apigenin, luteolin, orientin, quercetin, vitexin, and isovitexin); free amino acids (4-hydroxyisoleucine (0.09%), arginine, histidine, and lysine); calcium and iron; saponins (0.6-1.7%); glycosides yielding steroidal sapogenins on hydrolysis (diosgenin, yamogenin, tigogenin, neotigogenin); cholesterol and sitosterol, coumarin, fenugreekine, nicotinic acid, phytic acid, scopoletin, vitamins A, B1, C and nicotinic acid; and 0.015% volatile oils (n-alkanes and sesquiterpenes) [12].

The pharmacological properties of fenugreek are attributed to its various constituents. Fenugreek dialyzed seed extract has been reported to exert hypoglycemic effect by stimulating insulin signaling pathway resulting in activation of tyrosine phosphorylation of insulin receptor B, insulin receptor substrate 1 and p85 subunit of PI3-kinase in adipocytes and liver cells leading to GLUT4 translocation to the cell surface [13,14]. Fenugreek may exert its therapeutic effect through its alkaloids by modulation of insulin secretion [5]. The soluble dietary fiber (SDF) fraction of fenugreek significantly improved oral glucose tolerance in Type-1 and Type-2 DM. SDF fraction decreased intestinal disaccharidase activity and decreased glucose absorption, delayed gastric emptying and caused inhibition of glucose transport as the seeds contain around 50% pectin that forms a colloid suspension when hydrated and can decrease rate of gastric emptying and slow carbohydrate absorption [15]. The dietary fibers present in the fenugreek seeds, help in the management of metabolic abnormalities associated with diabetes such as peripheral insulin resistance and lipid abnormalities [16] and evidences of insulinotropic and anti-diabetic properties of 4-hydroxyisoleucine isolated from fenugreek seeds in glucose-dependent manner [17,18]. Further suggested that the anti-diabetic effect of 4-hydroxyisoleucine was, at least in part, from direct pancreatic beta cell stimulation. The furostanol saponins called trigoneoside Ia, Ib, IIa, IIb, IIIa, IIIb; glycoside and trifoenoside A were reported to be the active principles owing to their hypoglycemic effects [19].

The TC, low-density lipoprotein cholesterol (LDL-C) and triglyceride levels of the normal control Group 1 were significantly lower during 4^th^ and 8^th^ week than those of diabetic control Group 2 during the same period. The treatment Groups 3 through 7 showed significant decrease at the end of 4^th^ and 8^th^ week when compared to diabetic control (Group 2). The combination Groups 6 and 7 showed significant decrease among all the treated groups at the end of 4^th^ and 8^th^ week. All the groups revealed a significant increase in TC concentration at the end of 8^th^ week as compared to 4^th^ week. The HDL-C concentration of the normal control group was significantly higher than those of diabetic control throughout the experiment. The treatment Groups 3-7 showed a significant increase at the end of 4^th^ week when compared to diabetic control (Group 2). The Groups 6 and 7 showed significant increase among all the treated groups at the end of 4^th^ and 8^th^ week. All the groups revealed a significant increase in HDL-C concentration at the end of 8^th^ week as compared to 4t^h^ week (Table-3).

**Table-3:** Cholesterol concentration (g/dl) in different groups of rats.

Group	TC		HDL-C		LDL-C		TG	
			
	4^th^ week	8^th^ week	4^th^ week	8^th^ week	4^th^ week	8^th^ week	4^th^ week	8^th^ week
Non-diabetic control	80.27±1.02^aA^	92.09±1.19^aB^	36.57±0.89^e^	74.58±0.96^aA^	28.79±0.96^aA^	33.48±1.16^aA^	74.58±0.96^aA^	84.73±1.13^aB^
DM control	139.91±2.49^eA^	163.09±3.65^dB^	11.02±0.73^aA^	153.60±0.96^e^	98.17±2.50^fA^	117.44±4.56^eB^	153.60±0.96^e^	159.96±3.41^e^
DM+Insulin	103.40±1.68^cA^	120.53±1.88^cB^	24.49±0.71^bA^	114.75±0.89^dA^	55.96±1.75^dA^	74.46±1.52^dB^	114.75±0.89^dA^	114.34±2.56^bA^
DM+GM	100.91±1.74^cA^	124.48±2.05^cB^	26.82±1.09^cA^	107.31±0.65^cA^	52.63±2.09^dA^	76.54±1.98^dB^	107.31±0.65^cA^	116.39±1.99^bA^
DM+FG	113.40±1.34^dA^	123.35±2.60^cB^	23.51±0.46^bA^	110.67±1.95^cA^	67.76±1.58^eA^	65.34±2.37^cA^	110.67±1.95^cA^	127.37±2.68^cB^
DM+insulin+FG	89.77±1.19^bA^	103.58±2.91^bB^	30.67±0.38^dA^	96.88±1.52^bA^	39.73±1.31^cA^	46.59±3.06^bB^	96.88±1.52^bA^	98.14±0.73^dA^
DM+GM+FG	85.83±0.89^bA^	99.62±2.77^bB^	32.23±0.28^dA^	97.00±1.27^bA^	34.20±0.87^bA^	42.27±3.22^bB^	97.00±1.27^bA^	95.53±1.18^dA^

Values are mean±standard error (n=8). Means with different alphabets as superscripts differ significantly (p<0.05); Capital alphabets for horizontal comparison and small alphabets for vertical comparison. TC=Total cholesterol, HDLC=Highdensity lipoprotein cholesterol, LDLC=Lowdensity lipoprotein cholesterol, DM=Diabetic mellitus, FG=Fenugreek, TG=Triglyceride

**Figure-5:**
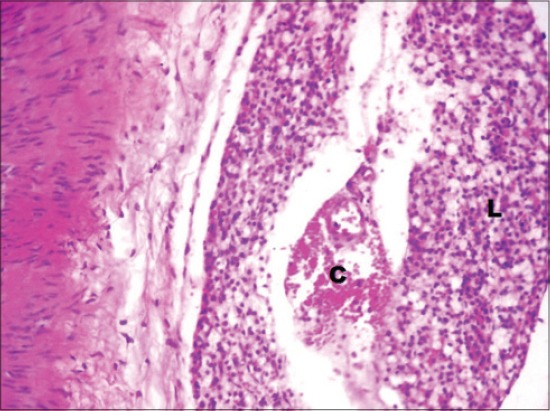
Photomicrograph of aorta showing marked congestion (C) in the tunica adventitia with heavy infiltration of lymphocytes (L). (H and E, ×200; Group 2).

**Figure-6:**
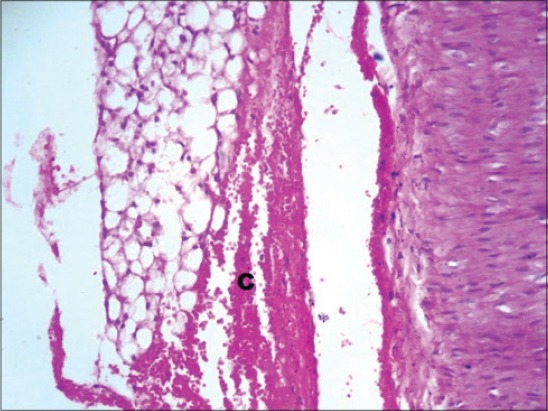
Photomicrograph of aorta showing marked congestion (C) between tunica layers (H and E, ×200; Group 3).

**Figure-7:**
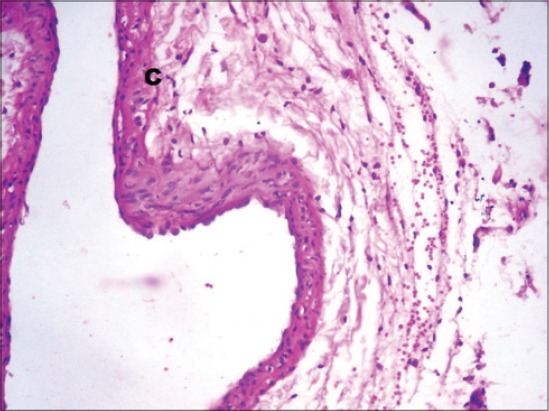
Photomicrograph of aorta showing mild congestion (C) in the tunica intima (H and E, ×200; Group 6).

**Figure-8:**
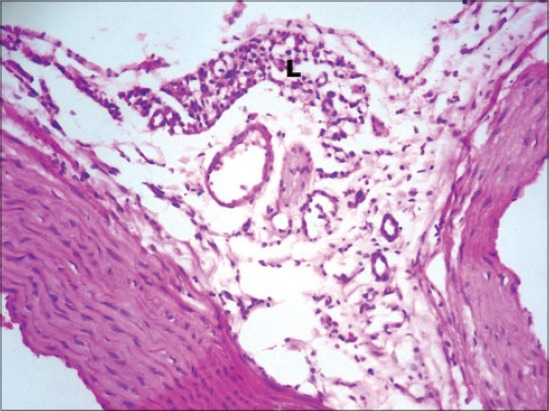
Photomicrograph of aorta showing mild infiltration of lymphocytes (L) (H and E, ×200; Group 7).

There was an increase in the levels of plasma TC, TG, LDL-C and reduced HDL-C in streptozotocin diabetic rats. The elevated levels of TC, TG, LDL-C and reduced HDL-C promote atherosclerosis and coupled with hyperglycemia-induced oxidative stress are the risk factors for cardiovascular disease. This increase is a result of increased breakdown of lipids and mobilization of free fatty acids from peripheral depots. Since insulin inhibits the hormone-sensitive lipase, the latter becomes active in DM. Excess fatty acids in serum or diabetic rats are converted into phospholipids and cholesterol in the liver. These two substances along with excess TG in the liver are discharged into the blood. Fenugreek treatment normalized the lipid profile due to its ability to stimulate insulin secretion [20]. This may be due to normalization of lipogenesis similar to the effect of insulin on lipid metabolism; also the fenugreek-induced normoglycemia caused no further degradation of accumulated lipid. In diabetes, the lipogenesis is decreased and lipolysis is increased releasing more free fatty acids into circulation, while fenugreek stimulates the hepatic lipogenic enzymes [5], thereby decreasing lipolysis. Galactomannan has been reported to exert a prominent selective inhibitory effect against intestinal lipase activity. It significantly delayed the absorption of LDL-C and TG and increased HDL-C [21]. The saponins present in fenugreek increased bile acid output due to increased conversion of cholesterol to bile acid by liver and prevented bile acid absorption, thereby increasing fecal weight and excretion of bile acids and cholesterol [22]. Glimepiride normalizes lipid profile by altering the lipid metabolizing enzymes and stimulating insulin secretion. Glimepiride alters the levels of lecithin: Cholesterol acyltransferase, lipoprotein lipase and cholesterol ester synthetase to near normal [9]. The results can be further supported by histopathological findings of the aorta, which showed marked congestion in the tunica adventitia with heavy infiltration of lymphocytes (Figure-5). In diabetic control Group 2. Moderate congestion was seen in the tunica intima in Groups 3, 4 and 5 (Figure-6), while mild congestion in the tunica intima was seen in Group 6 (Figure-7) and mild infiltration of lymphocytes was seen in Group 7 (Figure-8).

The total protein concentration of the normal control Group 1 was significantly higher during 4^th^ and 8^th^ week than those of diabetic control during the same period. The total protein concentration of Groups 3, 6 and 7 were comparable to that of Group 1 at the end of 4^th^ week. The Groups 3-7 showed significantly higher total protein concentration compared to Group 2 at the end of 8^th^ week. The albumin concentration of the normal control group was significantly higher during 4^th^ and 8^th^ week than those of diabetic control during the same period. The groups 3-6 showed significant increase in albumin concentration as compared to Group 2 at the end of 4^th^ week, while Group 7 value was comparable to that of Group 1 at the end of 4^th^ week. The Groups 3-7 showed significantly higher albumin concentration as compared to Group 2 at the end of 8^th^ week. The Groups 4, 6 and 7 were comparable to that of Group 1 at the end of 8^th^ week while the globulin concentration did not reveal any significant difference between groups. The A/G ratio of the normal control group was significantly higher throughout the experiment than those of diabetic control during the same period. All the treated Groups 3-7 were comparable to that of control Group 1 at the end of 4^th^ and 8^th^ week (Table-4). In DM, a variety of proteins are subjected to non-enzymatic glycation, and this is thought to contribute to the long-term complications of the disease [23]. The levels of serum total proteins were found to be decreased in this study. This decrease in diabetic rats may be ascribed to decreased amino acid uptake, greatly decreased concentration of variety of essential amino acids, increased conversion rate of glycogenic amino acids to carbon dioxide and water, and reduction in protein synthesis secondary to a decreased amount and availability of mRNA [24]. The fenugreek, insulin and glimepiride treatment reversed these changes in diabetic rats.

**Table-4:** Protein concentration (g/dl) in different groups of rats.

Group	Total protein (g/dl)		Albumin (g/dl)		Globulin (g/dl)		A/G ratio	
			
	4^th^ week	8^th^ week	4^th^ week	8^th^ week	4^th^ week	8^th^ week	4^th^ week	8^th^ week
Non-diabetic control	6.48±0.47^Ca^	8.08±0.22^eB^	3.20±0.06^Da^	4.41±0.16^dB^	3.28±0.48^aA^	3.67±0.40^aA^	3.28±0.48^aA^	3.67±0.40^aA^
DM control	2.97±0.37^aA^	5.38±0.09^aB^	0.82±0.10^aA^	1.79±0.09^aB^	2.16±0.36^Aa^	3.58±0.08^aB^	2.16±0.36^aA^	3.58±0.08^aB^
DM+insulin	5.45±0.30^bcA^	6.33±0.011^bB^	2.27±0.28^bA^	3.37±0.32^bcB^	3.18±0.30^aA^	2.99±0.28^aA^	3.18±0.30^aA^	2.99±0.28^aA^
DM+GM	4.89±0.07^bA^	6.78±0.19^bcB^	2.64±0.12^bcA^	3.54±0.20^bcB^	2.25±0.16^aA^	3.23±0.34^aA^	2.25±0.16^aA^	3.23±0.34^aA^
DM+FG	4.92±0.39^bA^	6.88±0.18^cB^	2.20±0.19^bA^	3.04±0.22^bB^	2.72±0.32^aA^	3.84±0.37^aB^	2.72±0.32^aA^	3.84±0.37^aB^
DM+insulin+FG	5.78±0.28^cA^	7.42±0.29^dB^	2.60±0.23^bcA^	3.98±0.09^cdB^	3.18±0.33^aA^	3.44±0.35^aA^	3.18±0.33^aA^	3.44±0.35^aA^
DM+GM+FG	6.16±0.45^cA^	7.06±0.15^cdA^	3.10±0.19^cdA^	4.21±0.09^dB^	3.06±0.38^aA^	2.85±0.17^aA^	3.06±0.38^aA^	2.85±0.17^aA^

Values are mean±standard error (n=8). Means with different alphabets as superscripts differ significantly (p<0.05); Capital alphabets for horizontal comparison and small alphabets for vertical comparison. DM=Diabetic mellitus, GM=Glimepiride, FG=Fenugreek, GM=Glimepiride

## Conclusion

The present study investigated that the pharmacodynamic interaction of fenugreek (herbal treatment) with insulin and glimepiride improved in the serobiochemical parameters in diabetic induced streptozotocin rats and showed a beneficial effect.

## Authors’ Contributions

CH: Designed and conducted the experiment under the guidance of AGR and YRR. BA helped in the analysis of various parameters in this experiment. CH and BA drafted and revised the manuscript. All authors read and approved the final manuscript.

## References

[1] Soud N.H.A, Khalil M.Y, Hussein J.S, Oraby F.S.H, Farrag A.R.H (2007). Antidiabetic effects of fenugreek alkaliod extract in streptozotocin induced hyperglycemic rats. J. Appl. Sci. Res.

[2] Yki-Jarvinen H (2001). Combination therapies with insulin in type 2 diabetes. Diabetes Care.

[3] American Diabetes Association (2002). Implications of the United Kingdom prospective diabetes study. Diabetes Care.

[4] Halberstein R.A (2005). Medicinal plants: Historical and cross-cultural usage patterns. Ann. Epidemiol.

[5] Neveen H.A.S, Khalil M.Y, Hussein J.S, Oraby F.S.H, Hussein F.A.R (2007). Antidiabetic effects of fenugreek alkaloid extract in streptozotocin induced hyperglycemic rats. J. Appl. Sci. Res.

[6] Ramesh B.K, Yogesh R.H.L, Kantikar S.M, Prakash K.B (2010). Antidiabetic and histopathological analysis of fenugreek extract on alloxan induced diabetic rats. Int. J. Drug Dev. Res.

[7] Dixit P, Ghaskadbi S, Mohan H, Devasagayan T.P (2005). Antioxidant properties of germinated fenugreek seeds. Phytother. Res.

[8] Reddy R.L.R, Srinivasan K (2009). Dietary fenugreek seed regresses preestablished cholesterol gallstones in mice. Can. J. Physiol. Pharmacol.

[9] Kakadiya J, Haresh M, Nehal S (2010). Investigation effect of glimepiride on diabetic marker and cardiac lipid parameter in isoproterenol induced myocardial infarction in diabetes in rats. Int. J. Ph. Sci.

[10] Singh U.B, Sulochana S (1997). Handbook of Histological and Histochemical Techniques.

[11] Kamalakkannan N, Prince P.S (2006). Antihyperglycaemic and antioxidant effect of rutin, a polyphenolic flavonoid, in streptozotocin-induced diabetic *Wistar* rats. Basic Clin Pharmacol. Toxicol.

[12] Ciftci O.N, Przybylski R, Rudzinska M, Surya A (2011). Characterization of fenugreek (*Trigonella foenum-graecum)* seed Lipids. J. Am. Oil Chem. Soc.

[13] Vijayakumar M, Govindrajan R, Rao C.H.C, Shirwaikar A, Mehrota S, Pushpangadan P (2006). Action of *Hygrophila auriculata* against streptozotocin-induced oxidative stress. J. Ethnopharmacol.

[14] Vijayakumar M.V, Pandey V, Mishra G.C, Bhat M.K (2010). Hypolipidemic effect of *fenugreek* seeds is mediated through inhibition of fat accumulation and up regulation of LDL receptor. Obesity (Silver Spring).

[15] Hannan J.M.A, Ali L, Rokeya B, Khaleque J, Akhter M, Flatt P.R, Abdel-Wahab Y.H.A (2007). Soluble dietary fibre fraction of *T. foenum graecum* (fenugreek) seed improves glucose homeostasis in animal models of type 1 and type 2 diabetes by delaying carbohydrate digestion and absorption and enhancing insulin action. Br. J. Nutr.

[16] Madar Z.L, Thorne R (1987). Dietary fiber. Prog. Food Nutr. Sci.

[17] Patel DK, Prasad S, Kumar K, Hemalatha S (2012). An overview on antidiabetic medicinal plants having insulin mimetic property. Asian Pac. J. Trop. Biomed.

[18] Haeri M.R, Izaddoost M, Ardekani M.R.S, Nobar M.R, White K.N (2009). The effect of fenugreek 4 – Ydroxyisoleucine on liver function biomarkers and glucose in diabetic and fructose-fed rats. Phytother. Res.

[19] Abdulaziz A, Yahya A (2013). Reproductive, cytological and biochemical toxicity of fenugreek in male Swiss albino mice. Afr. J. Pharm. Pharmacol.

[20] Yassin M, Mwafy S (2007). Protective potential of glimepiride and *Nerium oleander* extract on lipid profile, body growth rate, and renal function in streptozotocin-induced diabetic rats. Turk. J. Biol.

[21] Hamden K, Bassem J, Serge C, Abdallah A, El-Fazaa S, Najoua G, Abdelfattah E (2010). Potential protective effect on key steroidogenesis and metabolic enzymes and sperm abnormalities by fenugreek steroids in testis and epididymis of surviving diabetic rats. Arch. Physiol. Biochem.

[22] Stark A, Madar Z (1993). The effect of an ethanol extract derived from fenugreek (*Trigonella foenum-graecum*) on bile acid absorption and cholesterol levels in rats. Br. J. Nutr.

[23] Vlassara H, Li Y.M, Imani F, Wojciechowicz D, Yang Z, Liu F.T, Cerami A (1995). Identification of galectin-3 as a high-affinity binding protein for advanced glycation end products (AGE): A new member of the AGE-receptor complex. Mol. Med.

[24] Rajagopal K, Sasikala K (2008). Antihyperglycaemic and antihyperlipidaemic effects of *Nymphaea stellata* in alloxan-induced diabetic rats. Singapore Med. J.

